# Endocytic turnover of endothelial cell-membrane proteins as a driver of rat blood-brain barrier specialization and dysfunction

**DOI:** 10.1016/j.isci.2026.116231

**Published:** 2026-06-05

**Authors:** Alba Tomás-Sitjes, Gianluca Arauz-Garofalo, Marina Gay, Sònia Jarió, Marta Vilaseca, Valentina Schastlivaia, Maaike Kessen, Nicola Manicardi, Giuseppe Battaglia, Daniel Gonzalez-Carter

**Affiliations:** 1 Institute for Bioengineering of Catalonia (IBEC), Barcelona Institute of Science and Technology (BIST), Barcelona, Spain; 2 Institute for Research in Biomedicine (IRB Barcelona), Barcelona Institute of Science and Technology (BIST), Barcelona, Spain; 3 Catalan Institution for Research and Advanced Studies (ICREA), Barcelona, Spain; 4 Faculty of Science and Engineering, Maastricht University, Maastricht, the Netherlands

**Keywords:** Cellular physiology, Proteomics

## Abstract

The blood-brain barrier (BBB), formed primarily by specialized brain endothelial cells (BEC), is essential for nutrient transport, signal transduction, immune cell migration, and pathogen restriction. Although these functions are influenced by the identity and abundance of cell-membrane proteins, the role of protein endocytic turnover rate (ETOR, the dynamics of protein internalization, recycling, and degradation) governing BBB physiology remains poorly understood. Using *in vitro* proteomics, we analyzed ETOR across approximately 1,000 proteins in rat endothelial cells under healthy and pathological conditions to investigate BBB specialization and dysfunction. We found that BEC display a distinct ETOR profile that differentiates them from peripheral endothelial cells beyond membrane protein composition. Inflammatory conditions shifted the BEC ETOR profile towards a peripheral phenotype. Moreover, inflammation-induced abundance changes highlighted immune-response proteins, whereas ETOR alterations identified proteins associated with vascular remodeling. These findings establish ETOR as a dynamic regulator of BBB specialization and inflammatory dysfunction.

## Highlights

• Profiling membrane-protein endocytic turnover rates (ETORs) in endothelia
• ETOR profile drives BBB specialization independently of protein abundance
• Inflammation shifts BBB ETOR specialization towards a peripheral phenotype
• ETOR changes reveal inflammatory mechanisms missed by protein abundance shifts

## Graphical abstract

**Figure:**
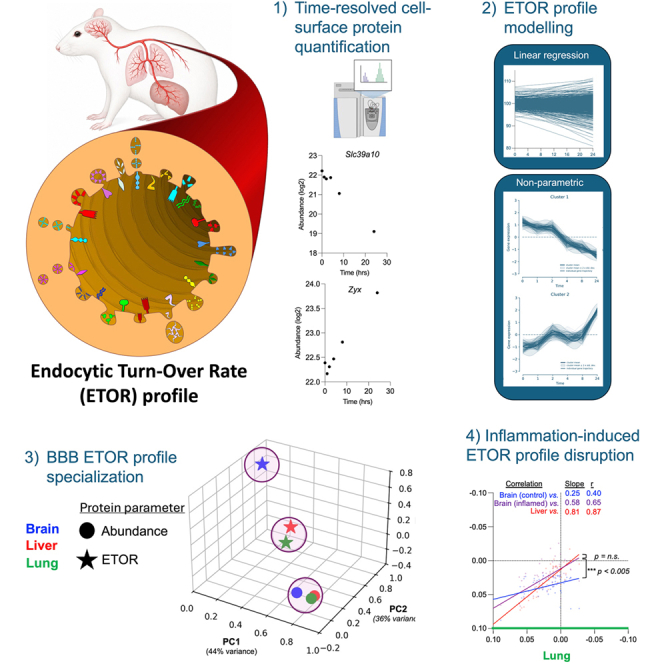

## Introduction

The blood-brain barrier (BBB), composed primarily of the highly specialized endothelial cells lining the cerebral vasculature, is a dynamic interface regulating the interaction between the blood circulation and the brain tissue to maintain the specialized microenvironment required for proper brain function. For instance, the BBB controls the transport of macromolecules to meet brain metabolic demands; responds to circulating cues to relay peripheral information to the brain; regulates the attachment and extravasation of circulating cells; and restricts the paracellular infiltration of toxins and pathogens. These functions are primarily mediated by proteins located on the cell membrane of brain endothelial cells (BEC). For instance, transcytosis receptors and transport channels such as TfR1 and Glut1 facilitate the transport of nutrients into the brain; signaling receptors such as Vegfr1 and Tie2 transmit peripheral signals to the brain; cell-adhesion proteins, including Icam1 and Vcam1, enable selective vascular attachment of circulating cells to regulate cell extravasation; and tight junction proteins such as Cldn5 and Cdh5 maintain the integrity of the BBB by restricting paracellular leakage.

While the abundance of cell-membrane proteins is known to dictate the function of endothelial cells, there is evidence that protein residency time on the cell-membrane (i.e., protein endocytic turnover rate, ETOR) acts as an important additional characteristic impacting cellular function. For example, increasing endocytosis of transport proteins facilitates nutrient delivery into cells.^1^^,^^2^ Additionally, endocytic internalization of signaling receptors influences vascular dynamics.^3^^,^^4^^,^^5^ Inhibiting endocytosis of cell-adhesion proteins facilitates the attachment and extravasation of leukocytes across endothelial cells.^6^ Furthermore, endothelial barrier impermeability is modulated by endocytic remodeling and membrane-residency half-life of cell-junction proteins.^7^^,^^8^^,^^9^ Therefore, ETOR serves as an important characteristic controlling the localization and function of cell-membrane proteins, thereby influencing endothelial cell behavior. However, it is currently unknown whether a specialized ETOR profile drives the phenotypic specialization of BEC to fulfill their unique functions, and whether pathological conditions modulate this ETOR profile to disrupt the BBB.

To explore these possibilities, we employ *in vitro* proteomics to quantify the ETOR of large arrays of individual cell-membrane proteins, thereby comparing ETOR profiles between rat endothelial cells from peripheral tissues (liver and lung) and the brain to detect phenotypic specializations. In addition, we examine how pathological conditions affect ETOR profiles in BEC to unravel novel mechanisms underlying BBB dysfunction during brain disorders.

## Results

### ETOR profile of brain endothelial cells

In order to establish the ETOR profile of rat BEC, the cell membrane abundance of labeled proteins was measured across time through affinity-capture quantitative proteomics. The quantification methodology employs a cell-impermeable biotinylating reagent to specifically label proteins on the cell membrane. The labeled proteins remaining on the cell membrane after specific endocytic internalization periods are captured through streptavidin-decorated agarose microbeads (Scheme S1).

**Figure 1:**
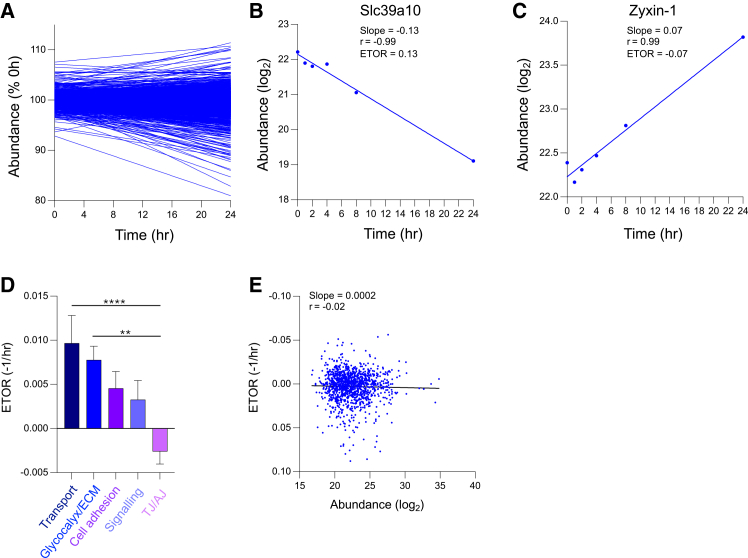
Quantification of endocytic turnover rates (ETOR) in rat brain endothelial cells *In vitro* proteomics were employed to quantify the abundance of labeled proteins (1184) on the cell membrane of brain endothelial cells across time (A). ETOR was quantified as the inverse of the resulting linear regression slopes (B and C). The biological relevance of the calculated ETOR was examined by grouping proteins according to biological function through bioinformatic filtering. Number of grouped proteins: Transport = 43; Glycocalyx/ECM = 171; Cell adhesion = 82; Signaling = 34; TJ/AJ = 92. Data are represented as mean ± SEM. Statistical analysis was assessed through a one-way ANOVA with *Tukey’s* post-hoc test. ∗∗∗∗, ∗∗*p* < 0.001, 0.01 *vs.* respective column (D). The relation of protein endocytosis to absolute abundance was examined by correlating ETOR with initial protein abundance (measured at 0 h) for individual proteins (E).

“True” cell membrane proteins were identified through bioinformatic filtering based on inclusion of selected gene ontology (GO) terms (Table S8, see section 4.6). A total of 1184 individual cell membrane proteins were analyzed, spanning a wide range of protein functions, including transporters, glycocalyx/extracellular matrix (ECM) proteins, cell adhesion molecules, signaling receptors, and cell-junction proteins (Table S1). Individual protein ETORs were calculated from the inverse of the slope of abundance change with time for each protein (Figures 1A–1C). Due to the relative quantification achieved through affinity-capture proteomics, abundance change could adopt either a negative slope (equating to a high ETOR) (e.g., the transporter protein Slc39a10, Figure 1B) or a positive slope (equating to a low ETOR) (e.g., the cell adhesion protein Zyxin-1, Figure 1C). To examine the biological relevance of the calculated ETOR values, proteins were grouped according to biological functions through GO term annotations (Table S9, see section 4.6). To avoid functional overlap, proteins belonging to a single “functional class” were included in the analysis (422 total individual proteins) (Figure 1D). Transport-associated proteins (e.g., LRP1, Slc39a10) had the highest ETOR, possibly reflecting the high nutrient transport demands of BEC. Conversely, proteins associated with cell-cell junctions (e.g., Jcad, Nf2) had the lowest ETOR, possibly reflecting the junctional stability necessary for the high paracellular impermeability of the BBB. Initial protein abundance (i.e., quantified at 0 h) was not correlated to ETOR (Pearson’s correlation coefficient, r = −0.02, Figure 1E), indicating endocytic profiles are not directly related to the cell membrane protein composition.

### ETOR profile specialization underlies BBB phenotype

**Figure 2:**
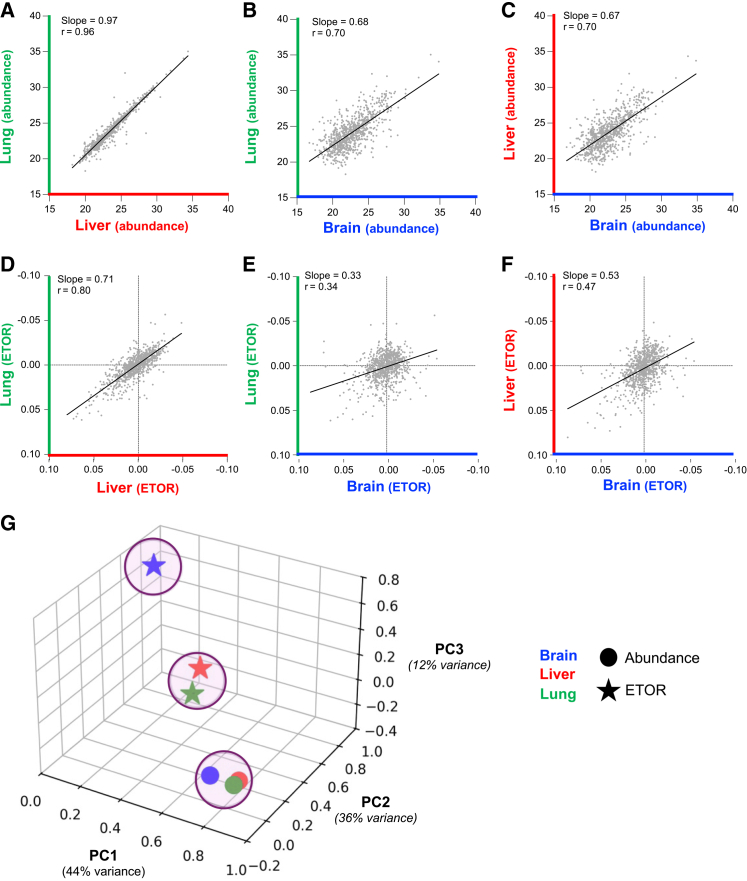
Comparison of protein abundance and ETOR profiles across endothelial phenotypes Protein abundance (at 0 h) (A–C) or ETOR (D–F) was correlated between lung *vs.* liver (A and D), lung *vs.* brain (B and E), and liver *vs.* brain (C and F) endothelial cells across 829 individual proteins shared between all three phenotypes. Principal component analysis (PCA) of all proteins across the six parameters was carried out to assess protein variability across phenotypes by clustering (DBSCAN) the principal component loadings for each parameter (G).

We next set out to examine whether the BBB phenotype conferred a specialized ETOR profile on BEC compared to peripheral endothelial cells. To that end, protein abundance change over time was quantified on endothelial cells extracted from the liver and lungs (Tables S2 and S3). The abundance and ETOR of individual proteins shared between all three phenotypes (829) were compared to assess the contribution of each parameter to phenotypic specialization. Comparison of individual protein abundance demonstrated a very strong correlation between liver and lung endothelial cells (r = 0.96, Figure 2A), indicating a highly conserved cell membrane protein composition in peripheral cells. Comparison against BEC demonstrated a strong correlation against both lung (r = 0.70, Figure 2B) and liver (r = 0.70, Figure 2C) endothelial cells, indicating cell membrane composition is relatively conserved across all three phenotypes. Interestingly, however, while ETOR profiles were still strongly correlated between peripheral endothelia (r = 0.80, Figure 2D), there was a marked loss of correlation in ETOR profiles between BEC and either lung (r = 0.34, Figure 2E) or liver (r = 0.47, Figure 2F) endothelial cells. To further explore the specialization of ETOR profiles on BEC, we carried out a principal component analysis (PCA) of the 829 proteins across the six parameters (“abundance” or “ETOR” in liver, lung, or brain EC) to quantify the component loading of each parameter, thereby assessing the contribution of each parameter to protein variability. The first three principal components were selected for analysis, as these captured >90% of data variability (Figure S1A). “Abundance” parameters for all three tissues had the strongest loading on PC1. Conversely, “ETOR” parameters for peripheral EC loaded mainly on PC2, while the “ETOR” parameter for brain EC loaded mainly on PC3 (Figures 2G and S1B–S1D). Clustering analysis of the component loading distributions demonstrated all three abundance parameters segregated into a single group, while peripheral ETOR parameters formed a separate group independent from the brain ETOR parameter (Figure 2G). Together, these data indicate that, while protein abundance variability is conserved between all three phenotypes, protein ETOR is specialized in BEC.

**Figure 3:**
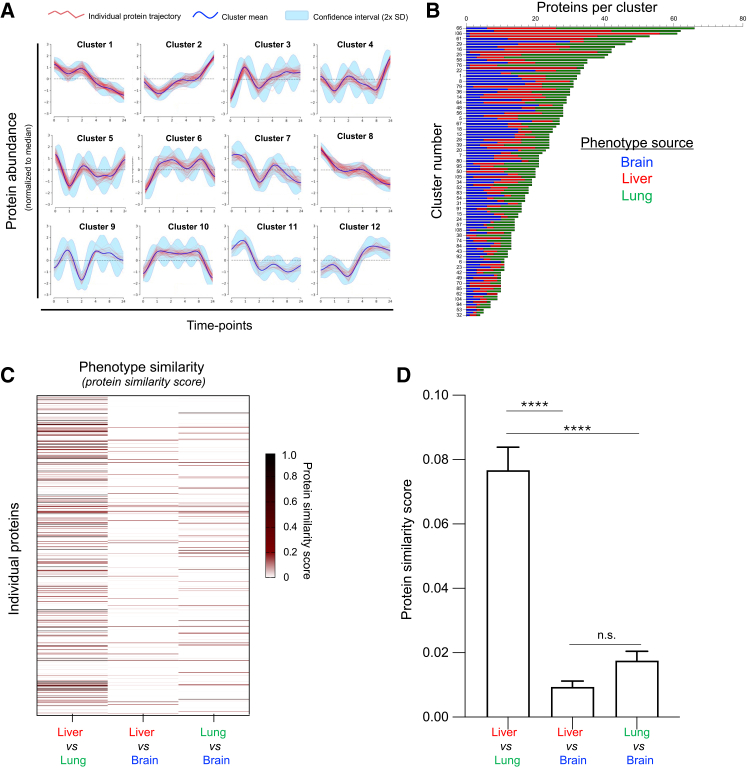
Non-parametric modeling of ETOR profiles across endothelial phenotypes Protein abundance change patterns were analyzed through an unsupervised, non-parametric Bayesian model (DPGP mixture model) to identify clusters of ETOR profiles. Whole-data analysis identified 110 individual clusters (A) (first 12 clusters depicted), containing proteins derived from each endothelial phenotype (B). Co-clustering probabilities (protein similarity scores) quantified the similarity of endocytic patterns between protein pairs (C). The average protein similarity score between endothelial phenotypes indicated phenotype specialization. Data are represented as mean ± SEM (D). Statistical analysis was assessed through a one-way ANOVA with *Tukey’s* post-hoc test. ∗∗∗∗, *p* < 0.001 *vs.* respective column.

The above data demonstrate ETOR calculations through linear regression modeling capture important biological characteristics of BEC contributing to their phenotypic specialization. In order to validate the linear modeling, we reanalyzed the proteomic dataset through an unsupervised, non-parametric Dirichlet Process Gaussian Process (DPGP) mixture model, allowing us to examine endocytic patterns without imposing a specific functional form on protein abundance change and intrinsically accommodating an unequally spaced time component.^10^ DPGP modeling of the proteomic dataset identified 110 individual clusters (Figures 3A and S2), containing proteins from each endothelial phenotype (Figure 3B; Table S10). The endocytic pattern similarity between pairs of proteins was calculated from their co-clustering probability (protein similarity score) (Figures 3C and S3; Table S11). The average similarity score between endothelial phenotypes allowed for the non-parametric comparison of phenotypic specialization. This analysis demonstrated a strong similarity in the ETOR profile between peripheral endothelial cells, with a clear differentiation from BEC (Figure 3D). Hence, the non-parametric modeling validated the ETOR values calculated by linear regression, confirming the phenotypic specialization of BEC.

### Inflammation disrupts brain endothelia ETOR specialization

**Figure 4:**
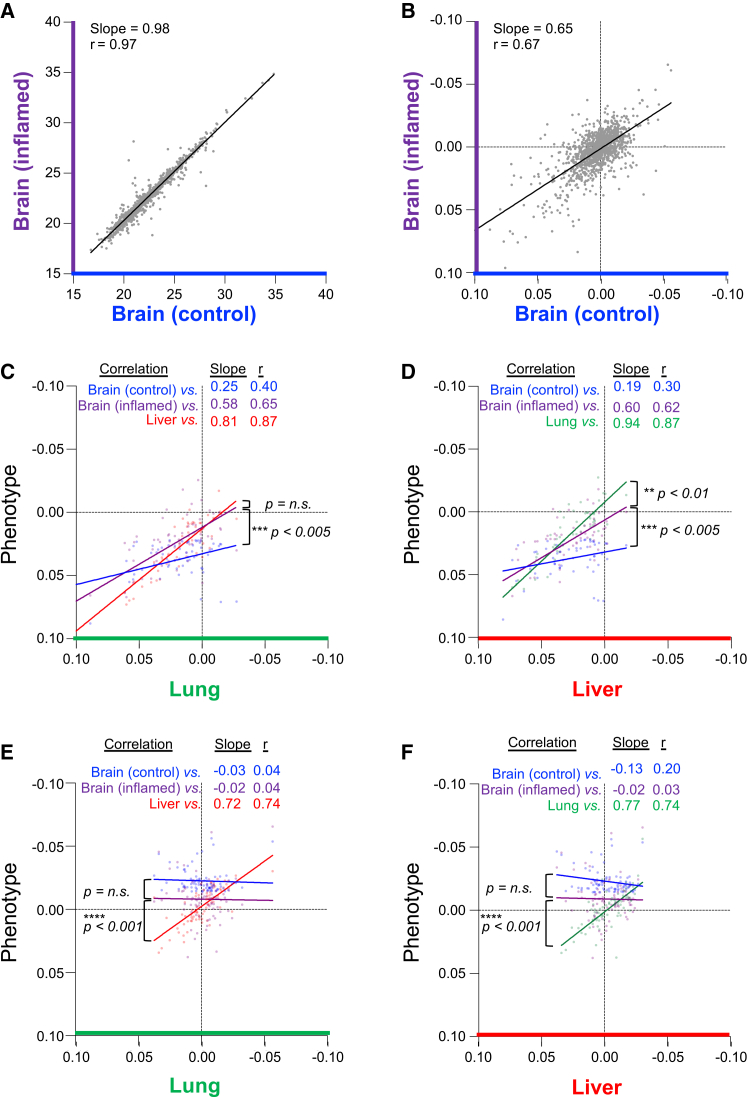
Effect of inflammation on the ETOR profile of brain endothelial cells The abundance (at 0 h) and ETOR of individual proteins (1150 shared proteins) were correlated between control *vs.* inflamed brain endothelial cells (A and B, respectively). To examine the inflammation-induced shift in correlation against peripheral endothelia, endothelia ETOR profiles were correlated *vs.* lung (C and E) or *vs.* liver (D and F) for proteins with high (C and D) or low (E and F) ETOR in “control” brain endothelial cells. Statistical difference was examined by comparing the regression slopes (F-test, GraphPad Prism) for each pair of correlations. *N.s.* indicates *p* > *0.05.*

Having established the contribution of ETOR towards the specialization of the BBB phenotype, we set out to examine how pathological conditions affected the ETOR profile of BEC. We selected inflammation as a pathological condition due to its importance in a wide range of brain disorders, from neurodegeneration to stroke. Inflammation was induced by treatment with lipopolysaccharide (LPS), which led to a marked production of pro-inflammatory markers (TNFα, nitric oxide) (Figure S4). In addition, membrane proteins known to be closely associated with vascular inflammation (e.g., Vcam1, Cd47, S1pr1)^11^^,^^12^^,^^13^ were detected in LPS-treated BEC but not in control BEC (Table S5). Despite the successful induction of inflammation, there was still a strong correlation in protein abundance between control and inflamed BEC (r = 0.97, Figure 4A). However, inflammation altered the ETOR profile of BEC, resulting in only a moderate ETOR correlation between control and inflamed BEC (r = 0.67, Figure 4B; Table S4). To examine whether the reduced ETOR correlation was due to a loss of the phenotypic specialization of BEC, we compared the ETOR profiles of control and inflamed BEC against the ETOR profile of peripheral endothelial cells. Comparing ETOR across all shared PM proteins (*c.* 860 proteins) demonstrated that inflamed BEC had a higher correlation *versus* either lung or liver endothelial cells (r = 0.48, 0.53, respectively) than control BEC (r = 0.36, 0.44, respectively) (Figures S5A and S5B). However, the increase in correlation was modest, with only the correlation slopes of {control BEC *vs.* lung} and {inflamed BEC *vs.* lung} being statistically different. However, the effect of inflammation was most strongly marked in proteins with high endocytic rates. Accordingly, comparison across proteins with the highest endocytic rates in control BEC (top 10% proteins) demonstrated a strong shift towards a peripheral ETOR profile under inflamed conditions (Figures 4C and 4D). Inflammation increased the ETOR correlation of brain *vs.* lung endothelial cells from r = 0.40 to r = 0.65. Furthermore, the correlation slopes of {inflamed brain *vs*. lung} and {liver *vs.* lung} became statistically equal (Figure 4C). The same trend of shifting the ETOR profile of inflamed BEC towards a peripheral ETOR profile was evident when comparing correlations *versus* liver endothelial cells (Figure 4D). Conversely, no significant differences in correlation *versus* peripheral endothelia were detected in the ETOR profiles of control and inflamed BEC when comparing ETOR across proteins with the lowest endocytic rates in BEC (bottom 10%) (Figures 4E and 4F). The effects of inflammation on protein abundance and ETOR were independent of each other, demonstrated by a very low correlation (r = 0.1) between ETOR change and abundance change (Figure S5C). In addition, LPS-treatment increased general endocytic rates in peripheral endothelial cells (Figure S6), further differentiating them from a healthy BBB endocytic profile.^14^

Together, the above data indicate that inflammation induced a loss of BBB specialization in the ETOR profile of BEC, shifting it towards a peripheral phenotype irrespective of changes in cell-membrane protein composition.

### Functional implications of ETOR modulation

**Figure 5:**
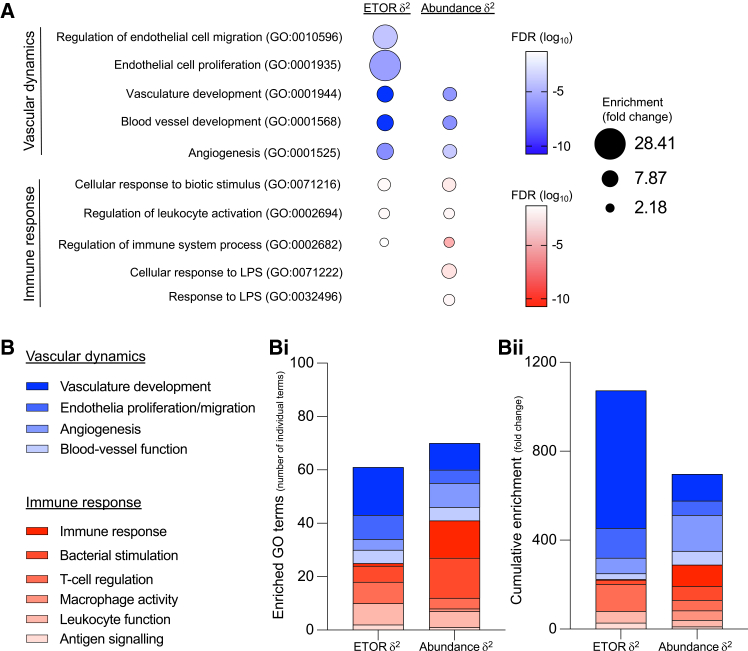
Inflammation-induced abundance or ETOR changes identify distinct protein sets involved in different biological functions Gene ontology enrichment analysis was used to assess the biological function of BEC proteins whose ETOR (“ETOR δ^2”^ protein set) or abundance (“abundance δ^2”^ protein set) is most affected by inflammation. Individual GO terms were categorized as “vascular dynamics” or “immune response” based on their biological process description. The enrichment (fold change) and false-discovery rate (FDR) are depicted for representative individual GO terms for each category (A). The total number of enriched GO terms (Bi) and the cumulative enrichment (Bii) for each category were quantified for all enriched GO terms.

Having established that inflammation disrupts the specialized ETOR profile of brain endothelia independently of changes in protein abundance, we set out to examine the functional implications of alterations in either protein ETOR or abundance. To this end, a GO enrichment analysis was carried out to identify biological processes overrepresented in the proteins with the greatest inflammation-induced changes in either ETOR or abundance (top 115 (10%) proteins by rank; protein sets termed ETOR δ^2^ or Abundance δ^2^, respectively). Intriguingly, a clear difference in biological processes appeared between the two protein sets (Tables S6 and S7). ETOR δ^2^ proteins had higher enrichment and lower false-discovery rates (FDRs) in GO terms associated with vascular dynamics, including “*angiogenesis”* (GO:0001525) and “*endothelial cell proliferation”* (GO:0001935) (Figure 5A). Conversely, Abundance δ^2^ proteins had higher enrichment and lower FDR in GO terms associated with immune response, including the “*regulation of immune system process”* (GO:0002682) and “*cellular response to LPS”* (GO:0071222) (Figure 5A). In total, ETOR δ^2^ proteins had a larger number of vasculature dynamics GO terms (Figure 5Bi), resulting in more than a doubling of cumulative enrichment of vascular dynamics biological processes compared to Abundance δ^2^ proteins (Figure 5Bii). While the differences in immune function were less marked, Abundance δ^2^ proteins still had a larger number of immune response GO terms (Figure 5Bi) and a higher cumulative enrichment of immune response biological processes (Figure 5Bii).

## Discussion

Endothelial cell-membrane proteins serve a wide range of functions, including barrier formation, nutrient uptake, signaling, and cellular transmigration. Together, they help define the endothelial phenotype. While the identity and abundance of proteins in the cell membrane are known to govern their function, there is evidence that the endocytic turnover of proteins is an important additional modulatory characteristic. In this work, we have developed an affinity-capture proteomic methodology to quantify the time-dependent changes in cell-membrane abundance of large arrays of cell-membrane proteins to examine the contribution of endocytic turnover rates (ETORs) to the phenotypic specialization of the BBB.

The biological relevance of the measured ETOR is supported by its alignment with protein function. As such, transport-related proteins and tight/adherens junction (TJ/AJ) proteins exhibited the highest and lowest ETOR, respectively, consistent with their roles in nutrient transport and intercellular stability. Although TJ/AJ and “cell-adhesion” proteins share nominally similar functions (i.e., cellular attachment to surfaces), their distinct ETOR values likely reflect fundamental differences in regulatory dynamics. TJ/AJ proteins primarily maintain stable intercellular barrier integrity, and their negative ETOR is consistent with prolonged membrane residency and limited basal endocytic turnover required for sustained BBB impermeability.^7^^,^^8^^,^^9^ In contrast, the broader cell adhesion category includes proteins involved in more dynamic processes such as leukocyte-endothelial interactions and cell migration requiring active turnover.^15^ Similarly, the relatively high ETOR of ECM/glycocalyx-associated proteins may reflect dynamic remodeling of ECM/glycocalyx-linked components at the endothelial surface,^16^ rather than structural instability. Together, these findings support ETOR as an indicator of protein turnover dynamics and cellular adaptability.

In addition to the biological relevance of ETOR, our analysis demonstrates that rat BEC possess a unique endocytic profile that differentiates them from peripheral endothelial cells over and above differences in the protein composition of the cell membrane. Furthermore, our analysis indicates that pathological conditions may shift the brain endocytic profile towards a peripheral phenotype, underlying the loss of BEC phenotypic specialization. These findings highlight that endocytic dynamics are an important characteristic that should be considered to achieve a deeper understanding of the unique features of the BBB and its dysfunction during disease. For instance, tracking changes in the endocytic rates of cell-junction and cell-adhesion proteins may shed light on barrier leakage and immune cell infiltration into the brain parenchyma, respectively, during disease progression.^17^^,^^18^^,^^19^ Similarly, examining changes in the endocytic rates of transporter proteins may deepen our understanding of the mechanisms controlling nutrient supply to the brain, for instance, during neurovascular coupling and circadian rhythms.^20^^,^^21^ Furthermore, changes in the ETOR of channel membrane proteins such as Slc39a10 may reflect a modulation in nutrient supply to the brain,^1^ potentially contributing to nutrient insufficiency associated with vasculature-associated brain disorders such as dementia.^22^

Furthermore, our analysis indicates that the endocytic rate of a protein is not directly related to its initial abundance. In line with this, we found that during inflammatory conditions, the proteins with the strongest change in abundance differ from the proteins with the strongest change in endocytic rates. Interestingly, each set of proteins was associated with a different biological response to inflammation, with proteins with the highest endocytic rate change being more associated with vascular dynamics, while proteins with the highest abundance change being more associated with immune response. This finding suggests that examining pathological effects solely through changes in protein abundance may overlook important pathological mechanisms executed by alternative proteins.

### Limitations of the study

Though our findings provide insightful details regarding the role of endocytic dynamics in BBB function, three important considerations need to be addressed. Firstly, while our labeling and capture methodology can accurately quantify the endocytic internalization of cell-membrane proteins with time,^14^ we are not able to discern the contribution of the two principal sorting pathways, namely lysosomal degradation and membrane recycling, to the dynamics of protein abundance. Indeed, our measurements will reflect the effective ETOR governed by three independent parameters: endocytosis rate, recycling rate, and degradation rate.^23^ Therefore, we are not able to ascertain which parameter contributes most to the differential ETOR between brain and peripheral endothelia, nor which parameter is most affected by inflammatory conditions.

Secondly, we have applied a stringent bioinformatic selection in our proteomic methodology to filter out non-PM proteins. This ensured analysis was restricted to proteins with a corroborated localization on the cell-surface, thereby avoiding proteins captured unspecifically during the extraction process. Previous PM-proteomic studies have demonstrated such unspecific capture of non-PM proteins is unavoidable due to the co-purification of intracellular PM interactor proteins.^24^ However, due to the limitations of bioinformatic annotations, the selection procedure inevitably led to the false negative designation of several proteins which have been predicted as cell-surface proteins by computational analysis (e.g., Ebp, Il1rap, M6pr, and Ssr1)^25^ or corroborated as brain-vascular surface proteins by *in vivo* capture methodologies (e.g., Abca9, Atp2a2, Colgalt1, and Pgap1).^26^ Indeed, recent studies have demonstrated that even proteins with exclusively nuclear/cytosolic localization bioinformatic annotations also have important cell-surface functions.^27^ Therefore, by limiting our analysis to “true” PM proteins, we may be overlooking proteins with important contributions to the endocytic dynamics of the cell-surface proteome.

Third, our measurements reflect the basal levels of endocytosis of cell-membrane proteins. Therefore, it will be of interest in future studies to examine how stimulus-dependent endocytic events (e.g., under the presence of specific ligands such as nutrients, signaling proteins, or circulating cells) alter the ETOR profile of each phenotype.

In conclusion, our study identifies the important role that endocytic dynamics of cell membrane proteins play in the specialized phenotype of BEC, highlighting ETOR profiles as an important cellular feature to understand more fully BBB physiology and mechanisms of disease progression.

## Resource availability

### Lead contact

Further information and requests for resources and reagents should be directed to and will be fulfilled by the Lead Contact, Dr. Daniel Gonzalez-Carter (daniel.gonzalezcarter08@alumni.imperial.ac.uk).

### Materials availability

This study did not generate new unique reagents.

### Data and code availability

• Data: All mass spectrometry proteomics data are available through the ProteomeXchange Consortium via PRIDE partner repository with the dataset identifier PXD067112. All data reported in this paper will be shared by the lead contact upon request.
• Code: Code developed for non-parametric analysis accessed through https://github.com/Molecular-Bionics-Labs/DP_GP_cluster_proteomics.
• Other items: Any additional information required to reanalyze the data reported in this paper is available from the lead contact upon request.

## Acknowledgments

We would like to thank Dr. Marius Kausas (ViaNautis Bio Ltd) for his kind guidance and assistance in the bioinformatic analysis. This work was supported by the Spanish State Research Agency (Agencia Estatal de Investigación, AEI) through a “Ramon y Cajal” research fellowship (RYC2022-036623-I) to DGC. Mass spectrometry/Proteomics was performed at the IRB Barcelona Mass Spectrometry and Proteomics Core Facility, which is granted in the framework of the 2014–2020 ERDF Operational Program in Catalonia, co-financed by the European Regional Development Fund (ERDF, IU16-015983).

## Author contributions

A.T.S., data curation, investigation, methodology, and writing-original draft; G.A.G., data curation, methodology, formal analysis, software, and writing-original draft; M.G., data curation, methodology, and software; S.J., data curation and methodology; M.V., project administration; V.S., data curation, formal analysis, and software; M.K., data curation, formal analysis, and software; N.M., investigation; G.B., project administration and supervision; D.G.C., conceptualization, formal analysis, funding acquisition, project administration, supervision, and writing-original draft. All authors reviewed and edited the final draft.

## Declaration of interests

All authors declare no conflicts of interest.

## STAR★Methods

### Key resources table

**Table:** 

REAGENT or RESOURCE	SOURCE	IDENTIFIER
sulfo-NHS-LC-biotin	Thermofisher	A39257
biotinylated protein interaction pull-down kit	Thermofisher	21115
Neutravidin-FITC	Thermofisher	A2662

**Biological samples**		

Primary rat lung endothelial cells	CellBiologics	RA6011
Primary rat liver endothelial cells	CellBiologics	RA6017
Primary rat brain endothelial cells	Isolated in house	–

**Deposited data**		

All mass spectrometry proteomics data are available through the ProteomeXchange Consortium via PRIDE partner repository with the dataset identifier PXD067112.	–	–

**Experimental models: Cell lines**		

Mouse brain endothelial cells b.End3.	American Type Culture Collection	CRL-2299

**Experimental models: Organisms/strains**		

Primary endothelial cells extracted from Sprague Dawley female rats (6–8 weeks old).	Charles River	–

**Software and algorithms**		

DIA by Neural Networks software (DIA-NN, v1.9.2). Code developed for non-parametric analysis accessed through https://github.com/Molecular-Bionics-Labs/DP_GP_cluster_proteomics	–	–

### Experimental model

Primary endothelial cells were extracted from the liver, lung and brain of 6–8 week-old Sprague-Dawley rats. When stated, immortalized murine brain endothelial cells (b.End3 cell line) were employed. Ethical approval (license) number: 25–018 (exp.12.03.2030).

### Method details

#### Endothelial cell extraction and culturing

Primary endothelial cells were extracted from the liver, lung and brain of 6–8 week-old Sprague-Dawley rats and cultured in coated wells to confluency before experimentation. Extraction and culturing protocols were identical to previously reported protocols. In brief, rat brain cortices (cleaned of meninges and visible blood vessels) were homogenized and digested with an enzyme mixture (trypsin, collagenase, dispase). Microvessels were separated from the digested tissue homogenate by centrifugation in a separation gradient buffer (25% v/v BSA). The resulting microvessel pellet was further digested with enzyme mixture and plated in culture flasks coated with collagen/fibronectin. Culturing was done in EGM-2 endothelial cell culture medium (with FBS, VEGF, FGF, IGF, EGF, ascorbic acid, hydrocortisone, gentamycin) (termed full-EGM), supplemented with puromycin (5 days @ 4 μg/mL, 3 days @ 1 μg/mL) to eliminate contaminating cells. Once a pure culture was obtained, cells were detached by trypsinization and plated on collagen/fibronectin-coated wells in full-EGM. Once cells reached 90% confluency, VEGF was removed from the culturing medium to promote a BBB phenotype by reducing para/transcellular permeability. Experimentation was carried out once monolayers reached 100% confluency (2–3 days following removal of VEGF).

#### Cell-membrane protein capture

Endothelial cell-membrane proteins were labeled with biotin through conjugation with the cell-membrane impermeable reagent sulfo-NHS-LC-biotin (thermofisher, A39257). Following incubation for specific time-points (in appropriate cell-culture medium, 37°C) to allow endocytosis, biotinylated proteins remaining on the cell-membrane were affinity-captured by conjugation to streptavidin-coated agarose microbeads. Following blocking of remaining available streptavidin sites on the bound microbeads with excess free biotin, cells were lysed (lysis buffer with 10% protease inhibitors). Lysates were collected into centrifugation filter tubes and microbead-captured biotinylated proteins separated from free proteins by through centrifugation according to the manufacturer protocol (Thermofisher, biotinylated protein interaction pull-down kit, cat no. 2115). Following washing of separated microbeads with acetate wash buffer (pH 5) and 0.5 M NaCl, microbeads were recovered in ammonium bicarbonate buffer (50 mM, pH 8) and frozen (−80°C) until mass spectrometry analysis.

#### On-bead tryptic protein digestion

Biotinylated proteins on thawed agarose microbeads were digested with trypsin and separated by centrifugation. Supernatant containing protein fragments was acidified (formic acid, 1%) and samples cleaned with C18 desalting spin columns (MonoSpin S type, GL Sciences) and subsequently with SCX tips following manufacturer protocols. Peptides were eluted with 5% ammonium hydroxide in 30% methanol, dried (Speed Vac) and stored at −20°C. For Liquid Chromatography-Mass Spectrometry (LC-MS) injection, dried eluates were reconstituted in 3% ACN/1% FA and loaded onto Evotips with an internal peptide standard mix (iRT kit, Biognosys) for MS quantification.

#### LC-MS/MS procedure

Mass spectrometry was performed on an Orbitrap Eclipse Tribrid mass spectrometer (ThermoFisher Scientific, San Jose, CA) connected to an EVOSEP One (EVOSEP, Odense, Denmark) via a nanoEasy Spray Source interface with a stainless-steel emitter (EV-1086 EVOSEP). Tryptic peptides were loaded onto the EVOTIP (EV-2013 EVOSEP) following the manufacturer’s instructions. The analytical column was a 15 cm × 150 μm ID, with Dr. Maisch C18 AQ. 1.5 μm beads (EV-1137 EVOSEP), placed into a column oven at 40°C. The eluents were 0.1% formic acid in water and 0.1% formic acid in acetonitrile. The Evosep One method was 15 SPD (88 min gradient), and the flow rate 0.22 μL/min. The mass spectrometer was operated in data independent mode (DIA). Full MS1 scans were acquired in the Orbitrap with a scan range of 350–1200 m/z and a resolution of 120,000 (at 200 m/z). Automatic gain control (AGC) was set to a target of 1 × 10^6^ and a maximum injection time of 56 ms. MS2 spectra were acquired in DIA using 33 m/z variable windows with 0.5 m/z overlap. MS2 spectra were analyzed in the Orbitrap with a resolution of 30,000 (at 200 m/z). A higher energy collision induced dissociation (HCD) method (28% NCE) was applied with an AGC target of 5 × 10^4^ and a maximum injection time of 55 ms. Orbitrap Eclipse Tune Application 3.5.3890 and Xcalibur version 4.5.445.18 were used to operate the instrument and to acquire data, respectively.

#### LC-MS data analysis

Spectrum files were analyzed using DIA by Neural Networks software (DIA-NN, v1.9.2),^28^ searching against UniProt reference proteome of Rattus Norvegicus (retrieved on June, 2024) and common contaminants.^29^ An independent DIA-NN search was carried out for each tissue-specific data subset, with constant search parameters throughout. Protein N-terminal acetylation, methionine oxidation and methionine excision at protein N-terminal were accepted as variable modifications. The maximum number of tryptic missed cleavages and number of variable modifications allowed were 2 and 1, respectively. The m/z range for the precursors and the fragment ions were set to 350–1200 and 200–2000, respectively. The match between runs option and the heuristic algorithm for the protein inference were also enabled. The remaining DIA-NN parameters were kept at their default values. The R package ‘diann-rpackage’ was used to generate protein group MaxLFQ^30^ quantifications prior to filtering precursors by q-value below or equal to 0.01, and the resulting datasets were further pre-processed, analyzed and visualized in R. In particular, contaminant protein groups were filtered out and the resulting protein group quantification values were log2-transformed, normalized using the cyclic LOESS algorithm implementation included in the ‘limma’ R package, and filtered requiring at least two peptides per protein group. Protein abundance was quantified and plotted for each time-point (0, 1, 2, 4, 8, 24h) and ETOR calculated from the inverse of the slope. All results comparing ‘Abundance’ and ‘ETOR’ employed the abundance of the 0 h time-point.

#### Bioinformatic filtering

The GO terms extracted from UniProt for each protein were employed to filter relevant proteins of interest. Plasma membrane (PM)-associated proteins (including glycocalyx and extracellular matrix proteins) were selected from all detected proteins based on inclusion of at least one GO term from a list of selected PM-relevant GO terms (Table S8). The stringent bioinformatic filtering ensured analysis was restricted to well-established PM proteins.

Protein function (applied in Figure 1D) was classified based on the inclusion of at least one GO term from curated lists of functional GO terms (Table S9). To avoid overlap in function, proteins were annotated only if GO terms exclusively from one functional list were included.

#### Principal component analysis

PCA was carried out on normalized data (mean = 0, SD = 1) of six parameters across 829 samples. The first 3 PC encompassed >90% of the data variance. A density-based clustering non-parametric algorithm (DBSCAN, eps = 2, min_samples = 1) was applied to determine the spatial clustering of loadings for three principal components of each parameter in a 3D space. Analysis was carried out in GraphPad Prism 11.

#### Gene ontology enrichment analysis

Enrichment analysis of biological process GO terms in specified protein lists was carried out through the GO Consortium analysis interface of the PANTHER classification system (PANTHER overrepresentation test), setting the *rattus norvegicus* database as reference list. GO terms were selected based on a False Discovery Rate <0.05 (Fisher’s exact test).

#### Non-parametric ETOR profile analysis

Unsupervised, non-parametric Bayesian modeling of protein abundance change patterns was carried out through a Dirichlet Process Gaussian Process (DPGP) mixture model^10^ (github.com/PrincetonUniversity/DP_GP_cluster) of the unfiltered protein dataset. Full description of the model may be found in the supplementary methods. In brief, the DPGP mixture model clusters protein abundance change patterns without requiring the number of clusters or trajectory shapes to be defined *a priori* (i.e., both are inferred from the data). Temporal endocytic trajectories within each cluster are modeled using a Gaussian Process with a composite kernel, allowing the model to capture a wide range of complex behaviors and intrinsically accommodating uniquely spaced sampling intervals. Cluster assignments are governed by the Dirichlet Process prior. Inference is performed via Gibbs sampling (Neal’s algorithm modification). Posterior samples are then used to construct a posterior protein similarity matrix, calculating the probability that a pair of proteins are assigned to the same cluster (co-clustering) across sampling iterations. This posterior co-clustering probability is used as a protein similarity score (PSS). The PSS between specific proteins derived from different endothelial phenotypes is employed to assess the phenotypic specialization of ETOR profiles.

#### Inflammation induction

Endothelial cells were treated with the bacterial cell wall component lipopolysaccharide (LPS) 22 h before ETOR profiling as above. Assessment of inflammation was carried out through quantification of nitric oxide through a Griess assay and the pro-inflammatory cytokine TNFa through an enzyme-linked immunosorbent assay. All brain endothelial experiments were carried out with freshly extracted primary brain endothelial cells, except for quantification of TNFa which employed the brain endothelial cell line b.End3.

#### General endocytic rate measurement

The internalization of biotinylated cell-membrane proteins was employed to measure general endocytic rates as previously described.^14^ In brief, cell-membrane proteins were labeled with biotin-NHS-sulfo and their abundance at the cell membrane at increasing endocytosis time-periods (incubated at 37°C) quantified through binding with neutravidin-FITC. One-phase decay models were employed to calculate the endocytic half-life.

### Quantification and statistical analysis

All statistical analysis was carried out in GraphPad Prism 11 and Python 3. Mean comparisons were carried out through one-way ANOVA with *Tukey’s* post-hoc test, unless otherwise stated. Results are displayed as mean ± SEM, unless otherwise stated.

## Supplemental Information

Document S1. Scheme S1, Figures S1–S6, and Methods S1

Table S1. Individual protein ETOR values in the brain (control) endothelia

Table S2. Individual protein ETOR values in liver endothelia

Table S3. Individual protein ETOR values in lung endothelia

Table S4. Individual protein ETOR values in brain (inflamed) endothelia

Table S5. Proteins detected specifically in brain (inflamed) endothelia

Table S6. GO term enrichment in proteins with the strongest inflammatory ETOR change

Table S7. GO term enrichment in proteins with the strongest inflammatory abundance change

Table S8. GO term inclusion for TURE PM protein classification

Table S9. Grouping of ETOR by protein function

Table S10. Protein composition (PM TRUE proteins) of DPGP clusters

Table S11. Comparison of single-protein endocytic profiles between endothelial phenotypes
