# Supplementary material for: Endocytic turnover of endothelial cell-membrane proteins as a driver of rat blood-brain barrier specialization and dysfunction
Source: iScience. 2026 Jun 5;29(6):116231. doi: 10.1016/j.isci.2026.116231 (PMC13264249; doi:10.1016/j.isci.2026.116231)
Supplement: Document S1. Scheme S1, Figures S1–S6, and Methods S1 [file mmc1.pdf]

iScience, Volume *29*

Supplemental information

Endocytic turnover of endothelial cell-membrane proteins as a driver of rat blood-brain barrier specialization and dysfunction

Alba Tomás-Sitjes, Gianluca Arauz-Garofalo, Marina Gay, Sònia Jarió, Marta Vilaseca, Valentina Schastlivaia, Maaike Kessen, Nicola Manicardi, Giuseppe Battaglia, and Daniel Gonzalez-Carter

Supplementary Schemes and Figures

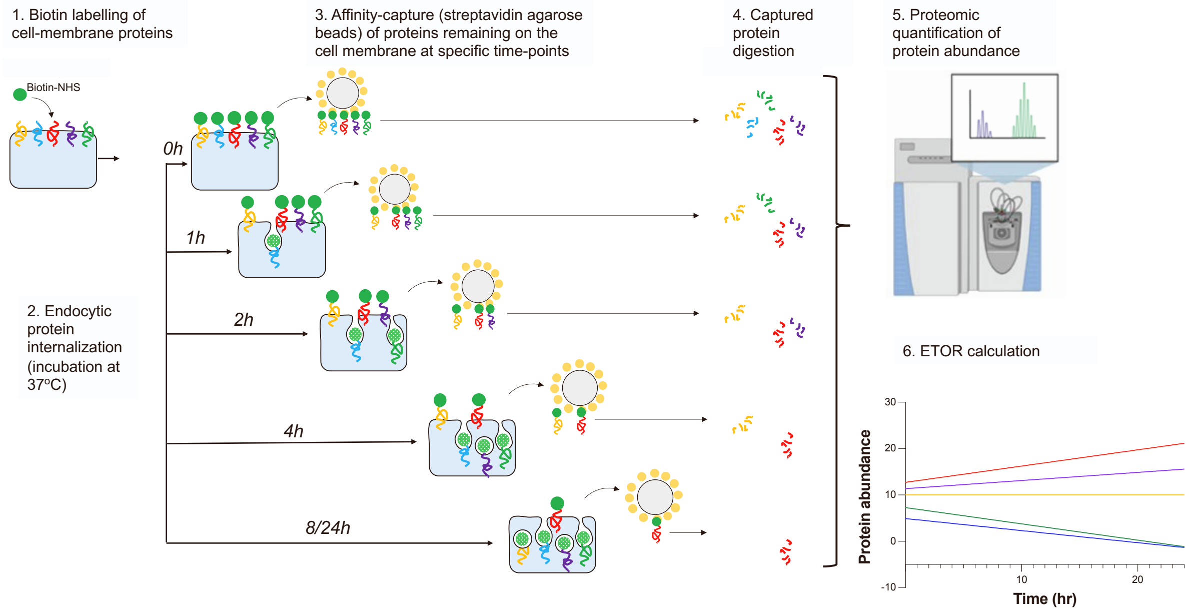

Scheme 1. Proteomic quantification of individual protein ETOR values. Step 1: Cell membrane protein biotinylation with biotin-NHS. Step 2: Increasing incubation time-periods (37ºC) to allow endocytosis of labelled proteins. Step 3: Affinity capture of biotinylated proteins remaining on the cell membrane by streptavidin-decorated agarose beads. As endocytosed proteins will compete less for streptavidin binding sites with proteins remaining on the cell membrane, there will be a time-dependent increase in non-endocytosed protein capture onto the beads (not shown in scheme). Step 4: Tryptic digestion of proteins captured on agarose beads. Step 5: Quantification of individual protein abundance by mass spectrometry. Step 6: Calculation of endocytic rate by protein abundance change over time.

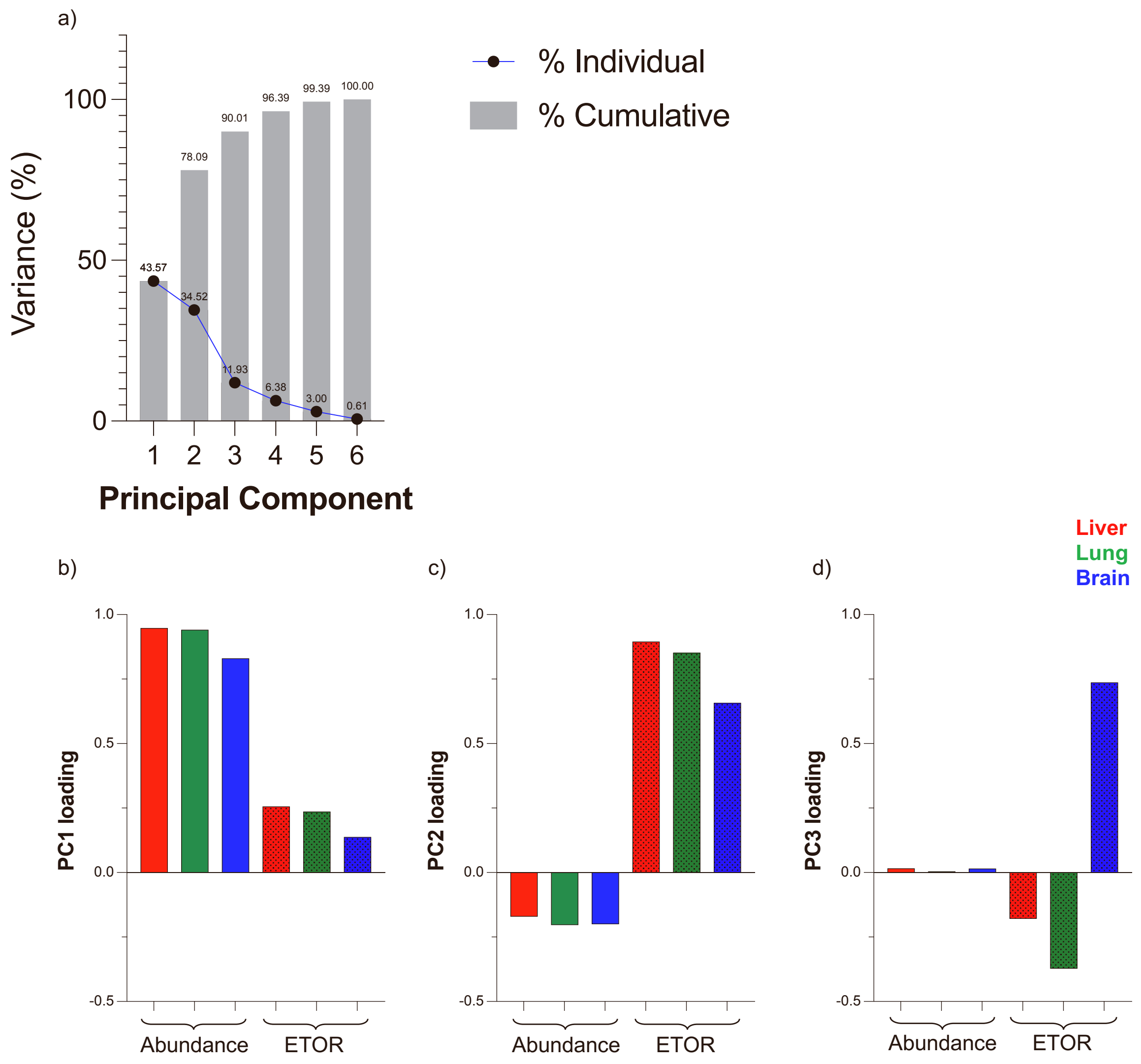

Supplementary figure 1. Principal component analysis values. Data variance capture by each principal component (a) and principal component loadings for each parameter (b).

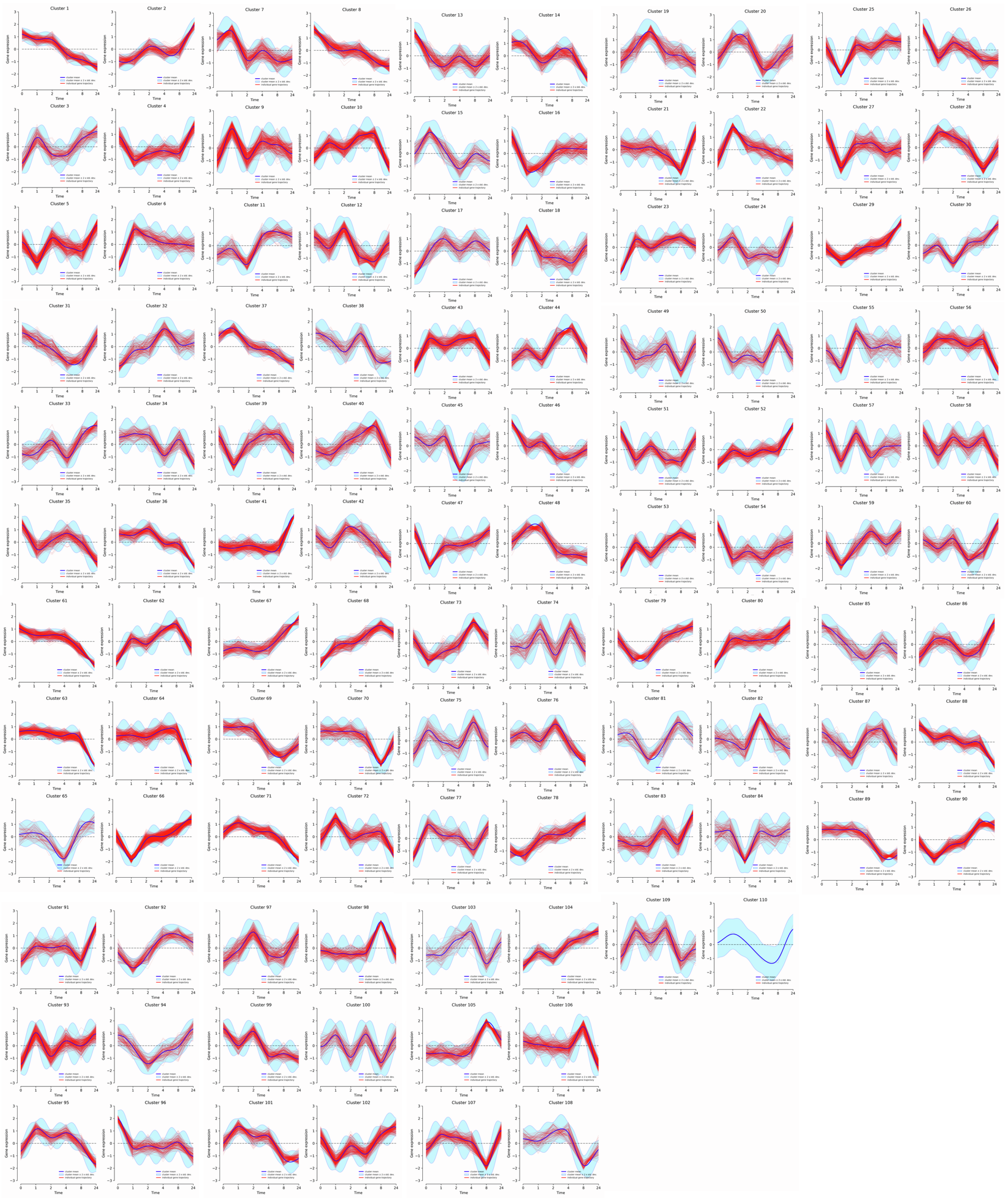

Supplementary figure 2. ETOR modelling through an unsupervised, non-parametric mixture model (Dirichlet Process Gaussian Process, DPGP). The DPGP mixture model allows for the classification of protein abundance change without *a priori* pattern assignment or prescription of numbers of clusters. As such, cluster assignment is derived directly from the data without assuming linearity and irrespective of the time-component. The DPGP process identified 110 individual clusters.

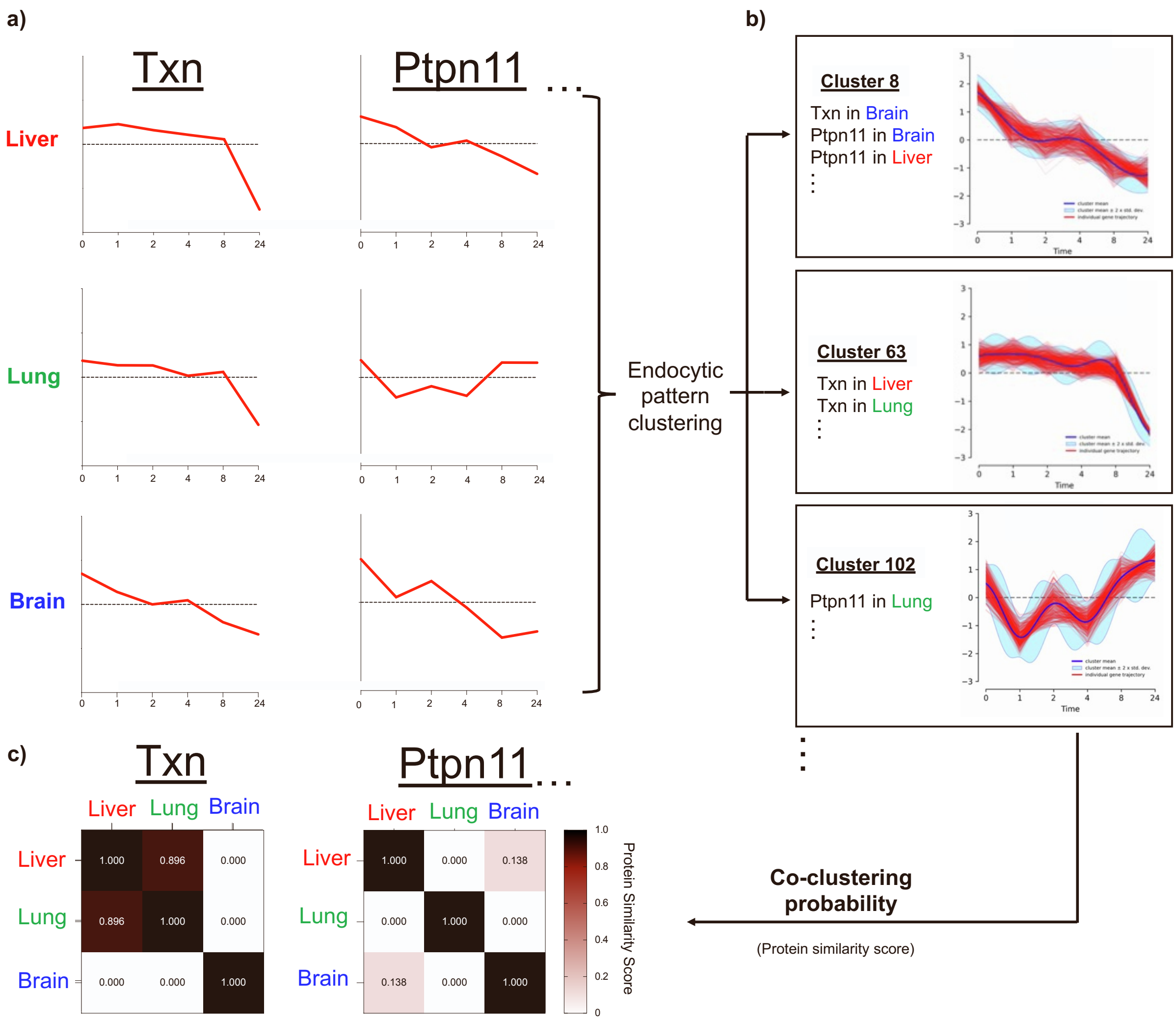

Supplementary figure 3. Endocytic profile similarity quantification through Dirichlet Process Gaussian Process (DPGP) non-parametric modelling. The probability that protein ETOR profiles (a) will be assigned to the same cluster (co-clustering probability) during the DPGP modelling (b) calculates the protein similarity score for specific protein pairs (c). Such protein similarity scores can then be employed to compare the similarity of ETOR profile of a single protein in two different endothelial phenotypes.

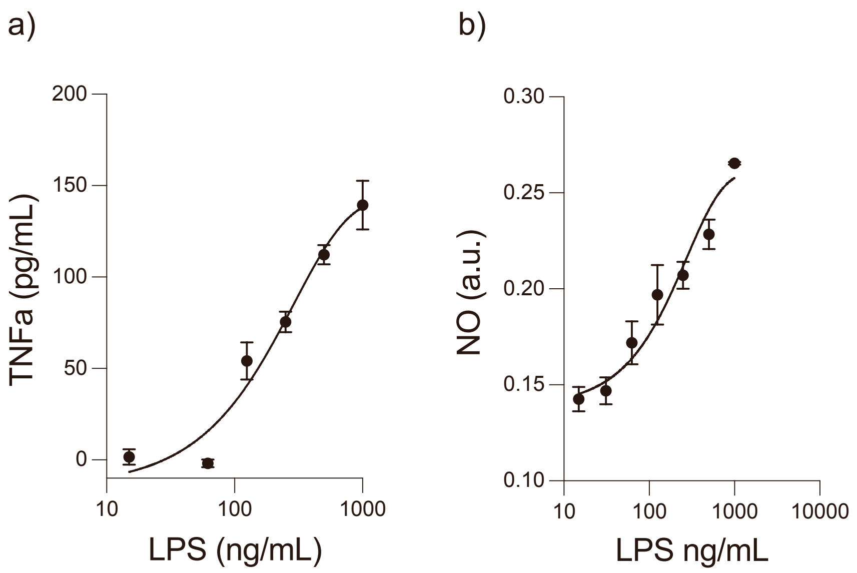

Supplementary figure 4. Inflammation induction in brain endothelial cells by treatment with lipopolysaccharide (LPS). TNFα quantification through ELISA in bEnd3 brain endothelial cells (a) and nitric oxide quantification through the Griess assay in primary brain endothelial cells (b). n = 2.

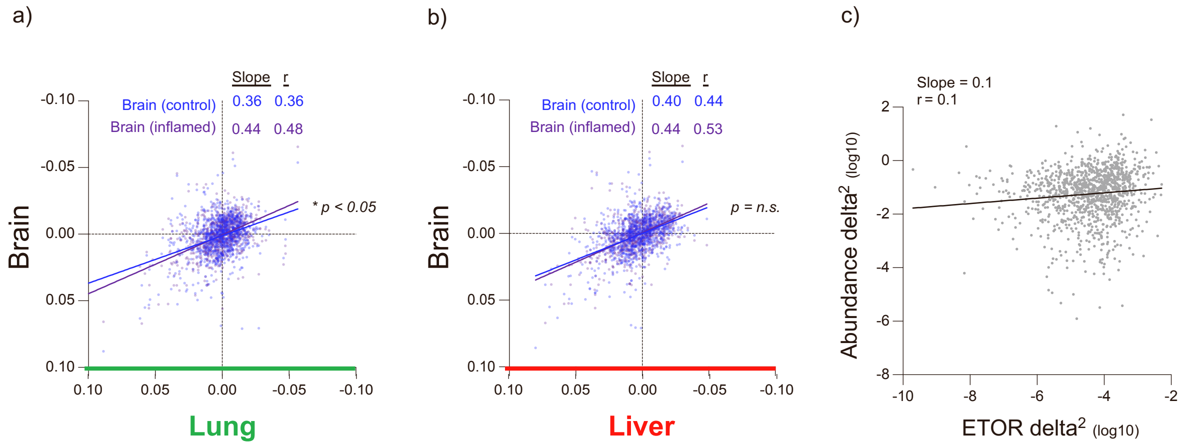

Supplementary figure 5. ETOR correlation across all proteins shared between brain (control), brain (inflamed), lung and liver endothelial phenotypes. ETOR correlation between {brain (control) *vs.* lung} and {brain (inflamed) *vs.* lung} (a). ETOR correlation between {brain (control) *vs.* liver} and {brain (inflamed) *vs.* liver} (b). Correlation between inflammation-induced changes in protein abundance and protein ETOR in brain endothelial cells (c). Statistical difference was examined by comparing the regression slopes (F-test) for each pair of correlations

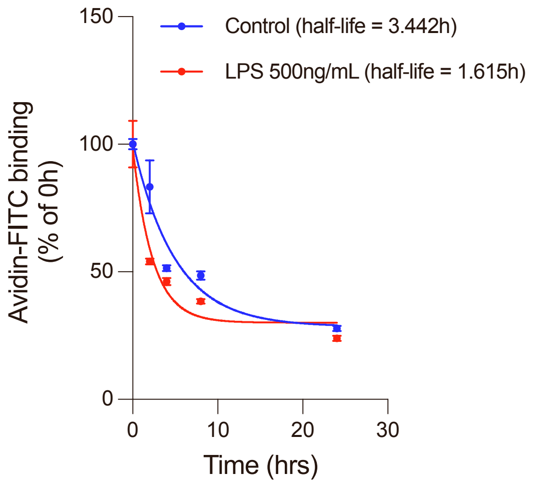

Supplementary figure 6. Quantification of general endocytic rates in control and inflamed primary liver endothelial cells. The internalization of biotinylated cell-membrane proteins was assessed by quantifying proteins remaining on the cell-membrane at increasing endocytosis time-periods (incubation at 37°C) by binding fluorescent (FITC) neutravidin.

Supplementary Methods S1

Non-parametric Bayesian clustering of endocytic profiles

To identify protein groups with similar endocytic profiles, a non-parametric Bayesian clustering framework was implemented, combining a Dirichlet Process (DP) for cluster discovery with Gaussian Processes (GPs) for modeling protein abundance trajectories. In contrast to traditional clustering methods such as k-means, this approach does not require *a priori* specification of the number of clusters, and in contrast to parametric approaches, does not assume a predefined functional form for the trajectories. Instead, both the number of clusters and the shape of the temporal trajectories are determined directly by the data.

The clustering framework follows the methodology described by McDowell et al (2018, doi: 10.1371/journal.pcbi.1005896) which integrates Dirichlet Process modeling for cluster determination with Gaussian Process regression for time-series biological data modeling. Cluster inference was performed using a modern Neal's Gibbs sampling algorithm for DP mixture models with non-conjugate priors. The protein abundance trajectories were modeled by GP with a composite kernel (linear combination of covariance functions) to capture the complex nature of the endocytic pattern.

The key components relevant to the present analysis are summarized below.

Dirichlet Process prior for cluster discovery

The Dirichlet Process defines prior distribution over cluster assignments and allows the number of clusters to be determined directly from the data.

For protein i, the probability of assignment to cluster k is given by

(1)

$$P(z_i = k|z_{-i}, \alpha) = \begin{cases} \frac{n_k}{N-1+\alpha} & If\ cluster\ k\ is\ occupied \\ \frac{\alpha}{N-1+\alpha} & If\ cluster\ k\ is\ new \end{cases}$$

Where z_i is the cluster assignment for protein i, z_{-i} represents all cluster assignments except protein i, n_k is the number of proteins in cluster k, N is the total number of proteins, and α is the concentration parameter controlling the propensity to create new clusters.

This formulation allows clusters to form dynamically and adjust cluster population during iterative inference, enabling flexible modeling of heterogeneous trajectory patterns.

Gaussian Process modeling of temporal trajectories

Protein abundance change trajectories were modeled using Gaussian Process (GP), allowing flexible non-parametric regression.

For cluster k, the GP is defined by a mean function $\mu_k(t)$ and covariance function $K_k(t, t')$. A composite kernel was used to capture multiple temporal behaviors:

(2)

$$K(t, t') = K_{RBF}(t, t') + K_{Linear}(t, t') + K_{Bias}(t, t')$$

The Radial Basis Function (RBF) kernel models smooth temporal variation [4]:

(3)

$$K_{RBF}(t, t') = \sigma^2{}_f \exp\left(-\frac{(t-t')^2}{2l^2}\right)$$

Where $\sigma^2{}_f$ is the signal variance and l is the characteristic length scale.

The linear kernel captures global temporal trends, while the bias kernel models baseline abundance levels.

For protein i with abundance vector $y_i = [y_{i1}, y_{i2}, \dots, y_{iT}]$ against time points $t = [t_1, t_2, \dots, t_T]$, the likelihood of protein i belonging to cluster k is:

(4)

$$P(y_i|z_i = k, \theta_k) = \mathcal{N}(y_i|\mu_k, K_k + \sigma^2{}_n I)$$

Where μ_k is the cluster mean function evaluated at time points t, K_k is the covariance matrix, $\sigma^2{}_n$ is the noise variance, and I is the identity matrix.

This GP framework captures correlations between nearby time points, allowing flexible modeling of linear, nonlinear, or oscillatory trajectories while providing uncertainty estimates for the predicted profiles.

Model inference

Model inference was performed using Markov Chain Monte Carlo sampling.

Burn-in phase

The burn-in phase allows the Markov chain to converge toward the posterior distribution and reduces dependence on initial conditions. Two burn-in stages were used, based on observed changes in clustering behavior during early iterations:

(1) Phase I (iterations 1-480): initial exploration of clustering configurations, during which clusters frequently formed and disappeared.
(2) Phase II (iterations 481-960): stabilization of cluster assignments and optimization of model hyperparameters.

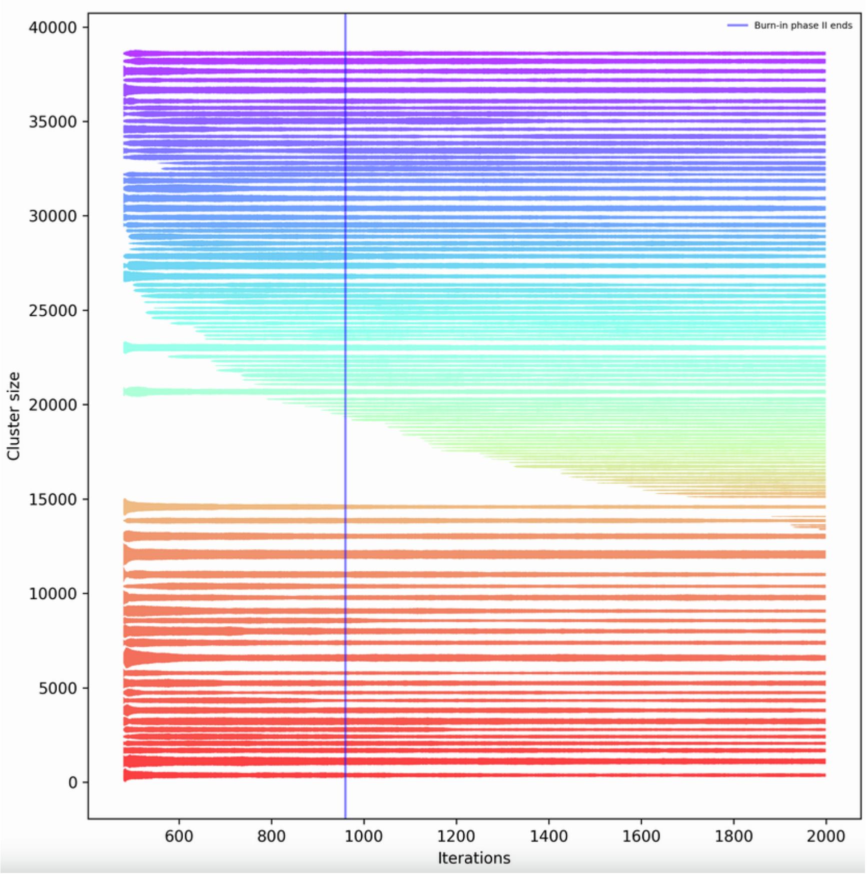

Sampling phase
Following burn-in, approximately 2000 sampling iterations were performed to take samples from the posterior distribution. Each iteration represents a possible clustering configuration, sampled with probability proportional to its posterior likelihood. Together, these samples characterize the posterior distribution over clustering solutions.
Posterior protein similarity matrix
To quantify similarity between proteins based on clustering results, a posterior similarity matrix S was constructed. Each matrix element represents the probability that two proteins co-cluster across posterior samples.
(5)

$$S_{ij} = \frac{1}{M}\sum_{m=1}^{M} \mathbb{I}({z_i}^{(m)} = {z_j}^{(m)})$$

Where M is the number of posterior samples, ${z_i}^{(m)}$ is the cluster assignment of protein i in sample m, and $\mathbb{I}(\cdot)$ is the indicator function. $S_{ij} \in [0,1]$ represents the probability that proteins i and j belong to the same cluster.
